# Supplementary material for: Nursing Students’ Knowledge Among Healthcare-Associated Infections: A Systematic Review
Source: Int J Environ Res Public Health. 2025 Oct 22;22(11):1609. doi: 10.3390/ijerph22111609 (PMC12652528; doi:10.3390/ijerph22111609)
Supplement: Supplementary file 1 [file ijerph-22-01609-s001.zip › S2. NIH Quality Assessment Tool for Observational Cohort and Cross-Sectional Studies.pdf]

Table 6. NIH Quality Assessment Tool for Observational Cohort and Cross-Sectional Studies

Criteria	**Colosi et al.,2011**[48]	**Alriyami et al.,2022** [34]	**Brosio et al., 2017** [35]	**Tavolacci et al., 2008**[32]	**D'Alessandro et al., 2014** [30]	**Majidipou et al., 2019** [31]	**Mazhar et al., 2025** [36]	**Bouget Mohammedi et al., (2025)**[33]
1. Was the research question or objective in this paper clearly stated?	**Yes**	**Yes**	**Yes**	**Yes**	**Yes**	**Yes**	**Yes**	**Yes**
2. Was the study population clearly specified and defined?	**Yes**	**Yes**	**Yes**	**Yes**	**Yes**	**Yes**	**Yes**	**Yes**
3. Was the participation rate of eligible people at least 50%?	**Not reported**	**Yes**	**Yes**	**Yes**	**Yes**	**Yes**	**Yes**	**Yes**
4. Were all the subjects selected or recruited from the same or similar populations (including the same time period)? Were inclusion and exclusion criteria for being in the study prespecified and applied uniformly to all participants?	**Yes**	**Yes**	**Yes**	**Yes**	**Yes**	**Yes**	**Yes**	**Yes**
5. Was a sample size justification, power description, or variance and effect estimates provided?	**No**	**Yes**	**No**	**No**	**No**	**No**	**No**	**No**
6. For the analyses in this paper, were the exposure(s) of interest measured prior to the outcome(s) being measured?	**N/A**	**No**	**No**	**No**	**No**	**No**	**No**	**No**
7. Was the timeframe sufficient so that one could reasonably expect to see an association between exposure and outcome if it existed?	**N/A**	**No**	**No**	**No**	**No**	**No**	**No**	**No**
8. For exposures that can vary in amount or level, did the study examine different levels of the exposure as related to the outcome (e.g., categories of exposure, or exposure measured as continuous variable)?	**No**	**No**	**No**	**Yes**	**Yes**	**Yes**	**Yes**	**Yes**
9. Were the exposure measures (independent variables) clearly defined, valid, reliable, and implemented consistently across all study participants?	**Yes**	**Yes**	**Yes**	**Yes**	**Yes**	**Yes**	**Yes**	**Yes**
10. Was the exposure(s) assessed more than once over time?	**No**	**No**	**No**	**No**	**No**	**No**	**No**	**No**

Criteria	Colosi et al.,2011[48]	Alriyami et al.,2022 [34]	Brosio et al., 2017 [35]	Tavolacci et al., 2008[32]	D'Alessandro et al., 2014 [30]	Majidipou et al., 2019 [31]	Mazhar et al., 2025 [36]	Bouget Mohammedi et al., (2025)[33]
11. Were the outcome measures (dependent variables) clearly defined, valid, reliable, and implemented consistently across all study participants?	**Yes**	**Yes**	**Yes**	**Yes**	**Yes**	**Yes**	**Yes**	**Yes**
12. Were the outcome assessors blinded to the exposure status of participants?	**Yes**	**No**	**No**	**No**	**No**	**No**	**No**	**No**
13. Was loss to follow-up after baseline 20% or less?	**N/A**	**Yes**	**Yes**	**Yes**	**Yes**	**Yes**	**Yes**	**Yes**
14. Were key potential confounding variables measured and adjusted statistically for their impact on the relationship between exposure(s) and outcome(s)?	**No**	**No**	**No**	**Yes**	**Yes**	**No**	**No**	**No**
**SCORE**	**6 /14**	**8/14**	**7/14**	**9/14**	**9/14**	**8/14**	**8/14**	**8/14**

Criteria	**Syed and Al-Rawi, 2024** [37]	**Shrestha et al., 2024** [38]	**Sharma & Bachani, 2023** [39]	**Thakker & Jadhav, 2015**[40]	**Darawad & Al-Hussami, 2013** [49]	**Wu et al., 2009** [50]	**Al-Rawajf]ah and Tubaishat 2013** [41]
1. Was the research question or objective in this paper clearly stated?	**Yes**	**Yes**	**Yes**	**Yes**	**Yes**	**Yes**	Yes
2. Was the study population clearly specified and defined?	**Yes**	**Yes**	**Yes**	**Yes**	**Yes**	**Yes**	Yes
3. Was the participation rate of eligible people at least 50%?	**Yes**	**Yes**	**Yes**	**Yes**	**Yes**	**Yes**	Not reported
4. Were all the subjects selected or recruited from the same or similar populations (including the same time period)? Were inclusion and exclusion criteria for being in the study prespecified and applied uniformly to all participants?	**Yes**	**Yes**	**Yes**	**Yes**	**Yes**	**Yes**	Yes
5. Was a sample size justification, power description, or variance and effect estimates provided?	**No**	**No**	**No**	**No**	**Yes**	**No**	No
6. For the analyses in this paper, were the exposure(s) of interest measured prior to the outcome(s) being measured?	**No**	**No**	**No**	**No**	**No**	**No**	No
7. Was the timeframe sufficient so that one could reasonably expect to see an association between exposure and outcome if it existed?	**No**	**No**	**No**	**No**	**No**	**No**	No

8. For exposures that can vary in amount or level, did the study examine different levels of the exposure as related to the outcome (e.g., categories of exposure, or exposure measured as continuous variable)?	**No**	**Yes**	**Yes**	**Yes**	**Yes**	**Yes**	Yes
9. Were the exposure measures (independent variables) clearly defined, valid, reliable, and implemented consistently across all study participants?	**Yes**	**Yes**	**Yes**	**Yes**	**Yes**	**Yes**	Yes
10. Was the exposure(s) assessed more than once over time?	**No**	**No**	**No**	**No**	**No**	**No**	No
11. Were the outcome measures (dependent variables) clearly defined, valid, reliable, and implemented consistently across all study participants?	**Yes**	**Yes**	**Yes**	**Yes**	**Yes**	**Yes**	Yes
12. Were the outcome assessors blinded to the exposure status of participants?	**No**	**No**	**No**	**No**	**No**	**No**	No
13. Was loss to follow-up after baseline 20% or less?	**Yes**	**Yes**	**Yes**	**Yes**	**Yes**	**Yes**	Yes
14. Were key potential confounding variables measured and adjusted statistically for their impact on the relationship between exposure(s) and outcome(s)?	**No**	**No**	**No**	**No**	**Yes**	**No**	No
**SCORE**	**8/14**	**9/14**	**9/14**	**9/14**	**10/14**	**9/14**	**7/14**

Criteria	**Ojulong et al., 2013** [42]	**Rahiman et al., 2018** [51]	**Blomgren et al.** [43]	**Khubrani et al., 2018** [44]	**Bello et al., 2011** [45]	**Mitchell et al., 2014** [46]	**Gould & Drey, 2013** [47]
1. Was the research question or objective in this paper clearly stated?	**Yes**	**Yes**	**Yes**	**Yes**	**Yes**	**Yes**	**Yes**
2. Was the study population clearly specified and defined?	**Yes**	**Yes**	**Yes**	**Yes**	**Yes**	**Yes**	**Yes**
3. Was the participation rate of eligible people at least 50%?	**Yes**	**Yes**	**No**	**Not reported**	**Not reported**	**No**	**Not reported**
4. Were all the subjects selected or recruited from the same or similar populations (including the same time period)? Were inclusion and exclusion criteria for being in the study prespecified and applied uniformly to all participants?	**Yes**	**Yes**	**Yes**	**Yes**	**Yes**	**Yes**	**Yes**
5. Was a sample size justification, power description, or variance and effect estimates provided?	**No**	**Yes**	**No**	**No**	**No**	**No**	**No**
6. For the analyses in this paper, were the exposure(s) of interest measured prior to the outcome(s) being measured?	**No**	**No**	**No**	**No**	**No**	**No**	**No**
7. Was the timeframe sufficient so that one could reasonably expect to see an association between exposure and outcome if it existed?	**No**	**No**	**No**	**No**	**No**	**No**	**No**

8. For exposures that can vary in amount or level, did the study examine different levels of the exposure as related to the outcome (e.g., categories of exposure, or exposure measured as continuous variable)?	**Yes**	**Yes**	**Yes**	**Yes**	**Yes**	**Yes**	**No**
9. Were the exposure measures (independent variables) clearly defined, valid, reliable, and implemented consistently across all study participants?	**Yes**	**Yes**	**Yes**	**Yes**	**Yes**	**Yes**	**Yes**
10. Was the exposure(s) assessed more than once over time?	**No**	**No**	**No**	**No**	**No**	**No**	**No**
11. Were the outcome measures (dependent variables) clearly defined, valid, reliable, and implemented consistently across all study participants?	**Yes**	**Yes**	**Yes**	**Yes**	**Yes**	**Yes**	**Yes**
12. Were the outcome assessors blinded to the exposure status of participants?	**No**	**No**	**No**	**No**	**No**	**No**	**No**
13. Was loss to follow-up after baseline 20% or less?	**Yes**	**Yes**	**Yes**	**Yes**	**Yes**	**Yes**	**Yes**
14. Were key potential confounding variables measured and adjusted statistically for their impact on the relationship between exposure(s) and outcome(s)?	**No**	**Yes**	**No**	**No**	**No**	**No**	**No**
**SCORE**	**8/14**	**10/14**	**7/14**	**7/14**	**7/14**	**7/14**	**6/14**
