# Supplementary material for: Nursing Students’ Knowledge Among Healthcare-Associated Infections: A Systematic Review
Source: Int J Environ Res Public Health. 2025 Oct 22;22(11):1609. doi: 10.3390/ijerph22111609 (PMC12652528; doi:10.3390/ijerph22111609)
Supplement: Supplementary file 1 [file ijerph-22-01609-s001.zip › S3. Data extraction.pdf]

Table 7. Data extraction

Authors (year)	Study type	Objective(s)	Geographic location (Country)	Final sample size	Eligibility criteria	Scales or tools	Main results	Effect estimate	Identified limitations
Colosi et al. (2011)[48]	Descriptive study	Investigate the level of knowledge of medical and nursing students on the prevention of HAI and measure differences between groups	Italy	n=117 (28 nursing)	a) Nursing students at Sapienza University; b) Enrolled in the final year	Standardized Infection Control Questionnaire (ICSQ) by Tavolacci et al., 2008	- Overall mean score: 17.63/25<br>- Nursing: 18.5 ±3.3<br>- Medicine: 17.4 ±3.9 (not significant)<br>- Better knowledge of standard precautions in both groups<br>- Poor knowledge of hand hygiene, especially alcohol-based rub use in both groups	Significant difference in hand hygiene area between nursing and medicine ($p=0.013$), in favor of nursing	- Small sample (pilot study)<br>- Administered only in the last week of the course<br>- No analysis of sources of knowledge<br>- Missing response rate 7.47%
Alriyami et al. (2022) [34]	Cross-sectional study	Examine and provide information on the level of knowledge of nosocomial infections among students and identify main sources of information	Asian	n=330 nursing students	a) Nursing students; b) ≥18 years; c) Completed at least one clinical course; d) Able to read/write in English; e) Consent to participate	Standardized Infection Control Questionnaire (ICSQ) by Tavolacci et al., 2008	- Average total correct score: 51.53% (SD=0.89), range 16–96<br>- Only 15.5% scored ≥70%<br>- Female students scored higher (53.84) vs males (45.03), $p<0.05$<br>- Main info sources: internet (46.4%), clinical experience (39.8%), curriculum (14.8%)	Significant differences in mean scores by gender ($p<0.05$) and source of knowledge ($p=0.001$, clinical experience associated with higher scores than curriculum and internet)	- Convenience sample from single university<br>- Limited generalizability

							- Most correct: alcohol-based rub as alternative to traditional washing (69.1%)<br>- Least correct: environment as main bacterial source (30.6%)		
Brosio et al. (2017) [35]	Cross-sectional study	Assess knowledge of Ferrara University nursing students on risk factors and most effective HAI prevention measures, focusing on hand hygiene practices and standard precautions; identify critical areas for training improvement	Italy	N=339 nursing students (59.9% 1st year; 25.9% 2nd year; 14.2% 3rd year)	a) Students of all three years; b) Nursing course at Univ. of Ferrara; c) Completed HAI training	Standardized Infection Control Questionnaire (ICSQ) by Tavolacci et al., 2008	Knowledge was sufficient only for Standard Precautions (SP, mean 8.9/10), and improved across years. Hand hygiene (HH) knowledge was sufficient only in third-year students (mean 7.0/10), while HAI knowledge remained insufficient across all years (mean 5.8/10). Awareness of the environment as a potential infection source and appropriate glove use for all patients were particularly low.-	-SP: 8.8 (1st), 9.1 (2nd), 9.2 (3rd); -HH: 6.7 (1st), 6.3 (2nd), 7.0 (3rd); -HAI: 5.5 (1st), 6.4 (2nd), 6.0 (3rd). Significant improvement across years in awareness of environmental infection sources ($p < 0.0001$), recognition of invasive procedures as risk factors ($p = 0.0117$), and knowledge on alcohol-based hand rub use vs traditional washing ($p < 0.05$).	- Only students present at lesson included (possible selection bias)<br>- Different participation rates by year<br>- Single-center study<br>- Questionnaire adapted but not validated for Italian context

Tavolacci et al. (2008)[32]	Cross-sectional study	Assess medical sciences students' knowledge on infection control and information sources	France	n=350 (78 nursing)	a) Students at University of Rouen; b) 1st year nursing; c) At least one clinical course	Standardized Infection Control Questionnaire (ICSQ)	Nurses had highest total score: 23.2 ± 2.35/30, higher than Physiotherapy (21.9), Medicine (21.1), Radiology Technicians (20.5) (P<.001)	Avg scores (whole cohort): Standard precautions 8.5/10, Hand hygiene 7.4/10, Nosocomial infections 5.7/10; nursing students highest in all areas	- Limited validity of knowledge test on nosocomial infections<br>- No standard reference measure<br>- Possible selection bias due to absenteeism<br>- No quantification of training hours
D'Alessandro et al. (2014)[30]	Cross-sectional study	Assess knowledge of medical and nursing students about HAI prevention	Italy	n=1461 (854 nursing)	a) Medicine 5th-6th year; b) Nursing 3rd year; c) Universities from 9 cities; d) Attended at least one hygiene/HAI prevention course	Standardized Infection Control Questionnaire (ICSQ) by Tavolacci et al., 2008	Acceptable score only in standard precautions; nursing total 18.6 vs medicine 17.4 (p=0.001); by area: SP=10.4; HH=5.3; HAI=2.9; significant differences (p<0.001)	Significant differences were found between medical and nursing students in all areas (p < 0.001), with a lower likelihood of adequate knowledge among medical students (OR = 0.54, p < 0.0001) and students aged ≥24 years (OR = 0.39, p < 0.0001).	- Information sources not investigated<br>- Possible variability in teaching programs
Majidipour et al. (2019)[31]	Cross-sectional study	Assess knowledge, attitudes, and practices (KAP)	Iran	n=102 students	a) Nursing students at Kermanshah University; b)	Self-administered structured questionnaire	- Good knowledge in 55.1%<br>- Positive attitudes in 53.8%	Previous training: OR=2.5 for good knowledge; Medical	- Possible social desirability bias<br>- Self-reported data

		of nursing science students on infection control			3rd or 4th year; c) Consent to participate	based on WHO & CDC guidelines	- Adequate practice in 48.9%<br>- Females higher performance scores (p=0.014)<br>- 3rd year better than 4th year (p=0.015)<br>- Direct correlation between knowledge and performance (r=0.46, p<0.0001)	students: OR=1.8 vs nursing	- Single-university sample<br>- Cross-sectional design prevents causality
Laiba Mazhar et al. (2025) [36]	Cross-sectional study	Assess knowledge and practices of standard precautions for infection control among BSN nursing students	Pakistan	n=64 nursing students	a) BSN students from private nursing college, convenience sampling	Questionnaire based on CDC & WHO guidelines	- 91% recognize importance of hand washing after contact; 86% understand standard precautions with HCV+ patients; 63% incorrectly believe necessary only for infected patients<br>- Good practices for hand washing (89%) and glove use (82%), low adherence to protective glasses (72%) and vaccination (75%)	Overall good knowledge (64% adequate) but critical gaps; generally good but inconsistent practices	- Small, non-representative sample<br>- Self-reported data with bias risk<br>- Cross-sectional design limits causal inference<br>- Possible non-response bias
Bouget Mohammedi S. et al. (2025)[33]	Quantitative observational pre-post study	Assess SP and HH knowledge before/after IPC training; identify predictors; assess	France	Pre-test=3,739 nursing students Post-	a) 1st-year nursing students; questionnaire before/after IPC	WHO HH questionnaire; Tavolacci SP questionnaire; teachers'	Initial moderate knowledge (35.67/50) improved to 37.55/50; NAD associated with higher scores; only	+2.1 points/50 (p<0.001); NAD increases odds 157-fold	- Selection bias

		teaching techniques effectiveness		test=2,378 students	training; b) informed consent	methods questionnaire	practical audit linked to significant improvement (p=0.050)		

Syed & Al-Rawi [37]	Descriptive cross-sectional study	To assess knowledge and hand hygiene practices among first-entry nursing students	Saudi Arabia	n = 304	a) Nursing students enrolled from 1st to 3rd year; b) Arabic language knowledge; c) Excluded students from other disciplines and non-Arabic speakers	Ad hoc questionnaire	- 94.2% recognized direct/indirect contact as the main route of HAI transmission; - 93.2% stated that handwashing prevents infections; - 83.2% washed hands before/after patient contact; - 59.9% used alcohol solution; - Female students scored higher in both knowledge (10.09 ± 1.27 vs 9.63 ± 1.48; $p = 0.004$) and practice (5.00 ± 1.25 vs 4.62 ± 1.46; $p = 0.037$)	Significant gender differences in knowledge ($p = 0.004$) and practice ($p = 0.037$), in favor of female students	- Single institution study; - Only junior Arabic-speaking students included; - No direct observation of practice; - Possible self-reporting bias
Shrestha et al. (2023) [38]	Descriptive cross-sectional study	To assess knowledge, perception, and confidence in IPC measures and analyze correlations among nursing students	Nepal	n = 163	a) Nursing students enrolled; b) With clinical experience; c) With IPC training before clinical practice; d) Excluded 1st-year B.Sc. Nursing students and those unwilling to participate	Ad hoc questionnaire	- Overall knowledge: fair (71%); - Best scores: general IPC principles (85%); - Poor knowledge: waste management (2%) and aseptic technique (52%); - General perception positive (mean 4.33/5); - Strong perception–confidence correlation ($r = 0.781$; $p < 0.001$)	Significant differences in knowledge by study level ($p < 0.05$). Correlations: knowledge–confidence ($r = 0.343$; $p < 0.001$), perception–knowledge ($r = 0.329$; $p < 0.001$)	- Sample from one university only; - Questionnaire did not cover all IPC areas; - Self-assessment did not measure actual compliance; - Possible bias from online completion without time limit

Sharma & Bachani (2023)[39]	Descriptive cross-sectional study	To assess knowledge, attitudes, practices, and perceived barriers to standard precautions among medical and nursing students	India (Madhya Pradesh)	n = 200 (100 medical, 100 nursing)	a) Enrolled medical and nursing students; b) Informed consent; c) Excluded unavailable/absent students	Ad hoc questionnaire	- Good knowledge: 57% nursing vs 48% medical; - Positive attitude: 63% nursing vs 56% medical; - Practice compliance: 42% nursing vs 38% medical; - Perceived barriers: lack of PPE, workload, insufficient training	Knowledge: 57% vs 48% ($p > 0.05$); Attitude: 63% vs 56% ($p > 0.05$); Practice: 42% vs 38% ($p > 0.05$)	- Single-center and small sample; - Possible self-reporting bias; - No direct observation; - Self-reported data may not reflect real behavior
Thakker & Jadhav (2015) [40]	Observational cross-sectional study	To assess and compare hand hygiene knowledge among medical, dental, and nursing students	India	n = 198 (84 medical, 74 dental, 40 nursing)	a) 2nd-year medical, dental, and nursing students; b) Informed consent; c) Excluded interns, residents, faculty, refusers	WHO Hand Hygiene Knowledge Questionnaire for Healthcare Workers	- 7.6% had good knowledge (>75% correct); - 69.2% moderate knowledge (50–74%); - 23.2% poor knowledge (<50%); - Critical gaps: minimum alcohol rub time (<40% correct) and correct method choice	Mean (±SD): Medicine 15.39 ± 3.13; Dentistry 14.09 ± 3.08; Nursing 13.57 ± 2.47. Median (IQR): Medicine 16 (14–18); Dentistry 15 (12–16); Nursing 13 (12–15). Significant differences: Medicine vs Dentistry ($p = 0.006$), Medicine vs Nursing ($p < 0.001$); Non-significant: Nursing vs Dentistry ($p = 0.068$)	- Single institution; - Small sample; - Self-administered (recall/self-assessment bias); - Cross-sectional (no causal inference)

Darawad & Al-Hussami [49]	Descriptive correlational cross-sectional study	To explore knowledge, attitudes, and compliance of Jordanian nursing students regarding infection control precautions	Jordan	n = 114	a) 3rd–4th year nursing students; b) With clinical training; c) Informed consent	Ad hoc questionnaire	Students reported insufficient knowledge: mean 49.64% (SD = 13.08; range 16–64%)	Mean = 49.64% (SD = 13.08; Range = 16–64)	- Single university; - Small sample; - Self-reported bias; - Cross-sectional design (no causal inference)
Wu, Gardner & Chang (2009)[50]	Cross-sectional study	To examine knowledge, application skills, and confidence of nursing students on standard and additional precautions	Taiwan	n = 175	a) 4th-year of 5-year nursing program; b) Willingness to participate	Infection Control Evaluation (ICE) – self-administered (knowledge, application, confidence)	- Low overall knowledge; - Better on standard precautions (sharps disposal 98.3%, mask/goggles 98.3%, vaginal secretions 95.4%); - Severe gaps in additional precautions (HIV 13.8%; mask with cough 13.2%)	Mean = 8.69/15 (SD = 1.55; range 3–12). 71% scored 8–10. Higher scores in students with 1-month training (p = 0.025). Paradoxically, no-training students scored higher than those with short courses (p = 0.017)	- Limited to southern Taiwan; - Convenience sampling; - Partially validated tool (knowledge reliable, application not tested); - Limited generalizability
Al-Rawajfah & Tubaishat (2015) [41]	Descriptive cross-sectional (web-based survey)	To assess knowledge and practices on standard precautions among Jordanian nursing students in	Jordan	n = 594	Nursing students enrolled in participating universities; no exclusion reported	Standard Precautions Questionnaire	- Good overall knowledge (mean 76.6% correct); - Gaps: tears 49.8%, sweat 62.1%, mask use in measles/chickenpox 58.8%	Mean = 13.8/18 (SD = 3.3; range 4–18). Quartiles: 25th = 13; 50th = 15; 75th = 16. Classification: 9.1% weak, 39.6% satisfactory, 51.3% excellent	- Online self-report bias; - Cross-sectional (no causal inference); - Limited generalizability to other contexts

		several universities							
Ojulong et al. (2013)[42]	Descriptive cross-sectional study	To assess knowledge and attitudes on IPC among health sciences students	Namibia	n = 385 (150 nursing students)	Medical, nursing, and health sciences students, University of Namibia	Ad hoc questionnaire	Nursing students: mean 64% correct; glove use 85%; sharps disposal 78%; hand hygiene 51%; mask use 44%. Lower scores than medical students	Significant difference vs medical students ($p < 0.05$)	- Single university; - Self-reported bias; - No analysis of learning sources
Rahiman et al. (2018)[51]	Descriptive cross-sectional study	To describe knowledge, attitudes, and practices on standard and transmission-based precautions among nursing students	South Africa	n = 301	2nd–4th year nursing students; excluded 1st years	Knowledge, Attitude and Practice of Standard and Transmission-Based Precautions Questionnaire	- Mean knowledge: 8.12 ± 1.7/10; - 47.4% good, 27.0% fair, 25.6% poor; - Best: standard precautions (60–90% correct); - Worst: transmission precautions (23% knew when to apply; 86% unaware of protective environment)	Overall mean = 8.12/10; 47.4% good; standard precautions 77% correct; transmission 52% correct	- Single institution; - Self-report bias; - Adapted questionnaire (not comparable with others)
Blomgren et al. (2024)[43]	Comparative descriptive study	To assess hand hygiene knowledge among nursing students (1st vs final semester) and nurses	Sweden	n = 201 nursing students	1st and final semester nursing students; registered nurses; volunteers	WHO Hand Hygiene Knowledge Questionnaire	- 1st semester mean 17.0 ± 2.1/25; - Final semester mean 18.8 ± 1.8/25; - Moderate knowledge overall (55.7%)	Significant difference between semesters (17.0 vs 18.8)	- Mix of students/nurses, limited data separation; - Self-report only; - Cross-sectional; - Limited representativeness (Uppsala)

Khubrani et al. (2018)[44]	Descriptive cross-sectional study	To assess knowledge of standard precautions and infection control among health sciences students	Saudi Arabia	n = 129 (14 nursing students)	Clinical-phase students in Medicine, Dentistry, Nursing, Pharmacy, Applied Medical Sciences	IPC Knowledge Questionnaire	Nursing students: 92.9% sufficient knowledge. Domains: general concepts 85.7%, hand hygiene 72.8%, PPE 73.8%, sharps 57.5%, HCW care 46.3%	Mean = 62.9 ± 7/100; sufficient ≥60%: 92.9%	- Very small nursing sample (n = 14); - Cross-sectional; - Self-report bias
Bello et al. (2011)[45]	Descriptive cross-sectional study	To assess and compare knowledge of nosocomial infections among health sciences clinical students	Ghana	n = 200 (70 nursing)	Clinical-phase students (Medicine, Nursing, Physiotherapy, Radiography; 1st–2nd clinical year)	Infection Control Standardized Questionnaire (ICSQ, Tavolacci 2008)	Nursing students: moderate knowledge (61.3% ± 2.3); - Best: standard precautions (79.9%); - Lowest: hand hygiene (56.6%), nosocomial infections (47.6%)	Total = 61.3% ± 2.3; domain range 47.6–79.9%; overall "moderate"	- Single institution; - Cross-sectional; - Social desirability bias; - ICSQ moderate reliability ($\alpha = 0.61$)
Mitchell et al. (2014)[46]	Multicenter descriptive cross-sectional study	To assess knowledge, intentions, and beliefs of final-year nursing students on IPC (standard and transmission-based precautions)	Australia (6 universities)	n = 349	Final-year nursing students	Online ad hoc survey	- Overall correct: 59.8% (95% CI 58.8–60.8); - Standard precautions 88.9% correct; - Transmission-based 27.2% correct	88.9% vs 27.2% ($p < 0.001$)	- Cross-sectional; - Online self-report bias; - Low response rate (21%); - Curricular differences not considered

Gould & Drey (2013) [47]	National online descriptive cross-sectional survey	To explore nursing students' IPC experiences during clinical placements	United Kingdom	n = 488	Preregistration nursing students, RCN members	Ad hoc online questionnaire	- All reported witnessing IPC non-compliance; - Most frequent: lack of hand hygiene (76.4%), jewelry (61.4%), nail polish/artificial nails (60%), isolation breaches (59.3%), PPE non-compliance (53.6%), poor sharps management (52.3%); - Theoretical IPC knowledge judged good	44–76% of students reported at least one non-compliance; overall good theoretical knowledge, but confusion on isolation/aseptic sequence	- Self-selected RCN members; - Completion 57%; - Perception-based, not direct testing; - Possible over-reporting bias
