# Supplementary material for: Nursing Students’ Knowledge Among Healthcare-Associated Infections: A Systematic Review
Source: Int J Environ Res Public Health. 2025 Oct 22;22(11):1609. doi: 10.3390/ijerph22111609 (PMC12652528; doi:10.3390/ijerph22111609)
Supplement: Supplementary file 1 [file ijerph-22-01609-s001.zip › S1. Joanna Briggs Institute Critical Appraisal Checklist.pdf]

Table 5. Joanna Briggs Institute Critical Appraisal Checklist

Author and Year	1. Inclusion criteria clearly defined?	2. Subjects and setting described in detail?	3. Exposure measured in a valid and reliable way?	4. Standard criteria used for measurement?	5. Confounding factors identified?	6. Strategies to deal with confounders stated?	7. Outcomes measured in a valid and reliable way?	8. Appropriate statistical analysis?	Score	%
Colosi et al., 2011 [48]	Yes	Yes	Yes	Yes	No	No	Yes	Yes	**6/8**	**75%**
Alriyami et al., 2022 [34]	Yes	Yes	Yes	Yes	Unclear	Unclear	Yes	Yes	**6/8**	**75%**
Brosio et al., 2017 [35]	Yes	Yes	Yes	Yes	No	No	Yes	Yes	**6/8**	**75%**
Tavolacci et al., 2008 [32]	Yes	Yes	Yes	Yes	No	Yes	Yes	Yes	**7/8**	**87,5%**
D'Alessandro et al., 2014 [30]	Yes	Yes	Yes	Yes	Yes	Yes	Yes	Yes	**8/8**	**100%**
Majidipour et al., 2019 [31]	Yes	Yes	Yes	Yes	Yes	Yes	Yes	Yes	**8/8**	**100%**

Mazhar et al., 2025 [36]	Yes	Yes	Yes	Yes	Unclear	Unclear	Yes	Yes	**6/8**	**75%**
Bouget Mohammedi et al., 2025 [33]	Yes	Yes	Yes	Yes	Yes	Yes	Yes	Yes	**8/8**	**100%**
Syed and Al-rawi, 2024 [37]	Yes	Yes	Yes	Yes	No	No	Yes	Yes	6/8	75%
Shrestha et al., 2024 [38]	Yes	Yes	Yes	Yes	No	No	Yes	Yes	6/8	75%
Sharma et al., 2023 [39]	Yes	Yes	Yes	Yes	No	No	Yes	Yes	6/8	75%
Thakker & Jadhav, 2015[40]	Yes	Yes	Yes	Yes	No	No	Yes	Yes	6/8	75%
Darawad & Al-Hussami, 2013 [49]	Yes	Yes	Yes	Yes	Yes	Yes	Yes	Yes	8/8	100%
Wu et al., 2009 [50]	Yes	Yes	Yes	Yes	Yes	Yes	Yes	Yes	8/8	100%

Al-Rawajfah et al., 2013 [41]	Yes	Yes	Yes	Yes	No	No	Yes	Yes	6/8	75%
Ojulong et al., 2013 [[42]	Yes	Yes	Yes	Yes	No	No	Yes	Yes	6/8	75%
Rahiman et al., 2018 [51]	Yes	Yes	Yes	Yes	Yes	Yes	Yes	Yes	8/8	100%
Blomgren et al. 2010[43]	Yes	Yes	Yes	Yes	No	No	Yes	Yes	6/8	75%
Khubrani et al., 2018 [44]	Yes	Yes	Yes	Yes	No	No	Yes	Yes	6/8	75%
Bello et al., 2011 [45]	Yes	Yes	Yes	Yes	No	No	Yes	Yes	6/8	75%
Mitchell et al., 2014 [46]	Yes	Yes	Yes	Yes	No	No	Yes	Yes	6/8	75%
Gould & Drey, 2013 [47]	Yes	Yes	Yes	Yes	No	No	Yes	Yes	6/8	75%
